# Veterinary Pharmaceutics: An Opportunity for Interprofessional Education in New Zealand?

**DOI:** 10.3390/pharmaceutics9030025

**Published:** 2017-07-26

**Authors:** Arlene McDowell, Rebekah Beard, Anna Brightmore, Lisa W. Lu, Amelia McKay, Maadhuri Mistry, Kate Owen, Emma Swan, Jessica Young

**Affiliations:** School of Pharmacy, University of Otago, Dunedin 9016, New Zealand; rebekahbeard1992@gmail.com (R.B.); anna.brightmore1@gmail.com (A.B.); Lisa.w.lu@live.com (L.W.L.); amelia2lily@gmail.com (A.M.); Maadhuri.mistry@gmail.com (M.M.); Kateowenz@gmail.com (K.O.); emma.kate.swan@gmail.com (E.S.); jessicajaimieyoung@hotmail.com (J.Y.)

**Keywords:** veterinary pharmacy, pharmacy education, interprofessional, New Zealand, One Health

## Abstract

Globally pharmacists are becoming increasingly involved in veterinary medicine; however, little is known about the level of interest for pharmacists playing a larger role in animal treatment in New Zealand. A key stakeholder in any progression of pharmacists becoming more involved in the practice of veterinary pharmacy is the veterinary profession. The aim of this study was to investigate views of veterinarians and veterinary students on the role of pharmacists supporting veterinarians with advice on animal medicines. Open interviews were conducted with veterinarians in Dunedin, New Zealand. Veterinary students at Massey University completed an online survey. Most veterinarians do not have regular communication with pharmacists regarding animal care, but believe it may be beneficial. In order to support veterinarians, pharmacists would need further education in veterinary medicine. Veterinary students believe there is opportunity for collaboration between professions provided that pharmacists have a better working knowledge of animal treatment. Most of the veterinary students surveyed perceive a gap in their knowledge concerning animal medicines, specifically pharmacology and compounding. While there is support for pharmacists contributing to veterinary medicine, particularly in the area of pharmaceutics, this is currently limited in New Zealand due to a lack of specialized education opportunities.

## 1. Introduction

The health and medical treatment of animals and humans are closely linked. Animal patients comprise a diverse range of species and can be broadly classified into the following groups; companion animals, livestock, captive animals and wildlife. Each of these groups of patients share some commonalities in health conditions as the human population and some unique to their respective life histories. For example, domestic pets are known to suffer from osteoarthritis and diabetes mellitus. An example of a condition that is unique to the life history of the animal is the case of the endemic Australian Tasmanian Devil (*Sarcophilus harrisii*) and its susceptibility to Devil facial tumor disease, a rare transmissible, parasitic cancer. The fatal infectious disease is spread during biting that occurs during social interaction in this species and low genetic diversity in wild populations has led to a population decline of >80% [1]. Diseases can also be transferred from animals to humans via zoonotic infections and it has been estimated that 61% of human diseases are caused by zoonotic pathogens [2] (e.g., HIV, SARS, Zika virus). The presence of reverse zoonosis (zooanthroponosis), where humans transmit pathogens (e.g., *Mycobacterium tuberculosis* and influenza A) to animals causing disease has also been highlighted [3]. The inter-dependence between the health of humans and that of animals has led to the development of the concept of One Health. This international initiative aims for a holistic approach to improving health that incorporates humans, animals and their shared environment [4]. The aim is that interdisciplinary collaboration between veterinarians, doctors and ecologists can find solutions to global health challenges. The knowledge and training pharmacists have and their accessibility in communities means that pharmacists are ideally placed to make a contribution to the One Health initiative.

Globally, pharmacists are becoming increasingly involved in veterinary medicine. Many veterinarians are now writing prescriptions for their patients instead of dispensing medicines in their practice. This has been driven by the increasing costs of medicines and the increasing use of common human medicines in animal care [5]. Veterinarians are also choosing to write prescriptions because they require drugs that they do not have in their practices in the appropriate strength or in a suitable formulation for their animal patients [6]. Consequently, the number of animal prescriptions being extemporaneously prepared and dispensed in pharmacies is increasing. When pharmacists dispense a medication for a human patient they have a legal and ethical obligation to ensure the dose, route of administration, medication, and frequency are all suitable for the intended use of the medicine to treat a specific condition. In order to make a meaningful contribution to this interprofessional relationship, both groups need the appropriate skills and knowledge to do so.

In New Zealand, the role of the pharmacist has not incorporated veterinary medicine to the extent of global contemporaries, with the roles of veterinarians and pharmacists remaining quite separate and defined [7]. The Pharmacy Council of New Zealand governs the practice of pharmacists and defines pharmacy practice to include the technical aspects of pharmacy services, preparation of pharmaceuticals and medicines supply management as well as patient-centered care with counseling, providing drug information and monitoring drug therapy [8]. According to the New Zealand Veterinary Association, veterinarians are involved in the diagnosis, treatment, and disease prevention of animals [9]. To be a veterinarian, one must hold a current practicing certificate and be registered with the Veterinary Council of New Zealand [10]. 

Previous authors have investigated the views and experiences of pharmacy students and pharmacists in relation to veterinary pharmacy [11,12,13]. The majority of those surveyed support the incorporation of a knowledge of veterinary medicine to enhance their practice. A key stakeholder in any progression of pharmacists becoming more involved in the practice of veterinary pharmacy is the veterinary profession. Consequently, the aim of this study was to investigate the views of veterinarians and veterinary students on the potential role of pharmacists supporting veterinarians with advice on animal medicines in New Zealand.

## 2. Materials and Methods

Ethical approval for this study was obtained from the School of Pharmacy, University of Otago (D13/287) prior to commencement of the study. Each participant was given information outlining the aim the project, participants and their role in the study. All subjects gave their informed consent for inclusion before they participated in the study.

The study was conducted using a mixed methods approach. Data was collected through focused discussions with veterinarians from the Dunedin region of New Zealand. Veterinarians were invited to participate in a semi-structured interview that took 15–20 min to complete. In total four veterinarians agreed to participate. The interview was based on 14 questions (Appendix A) and was asked by pairs of interviewers. Questions focused on the veterinarian’s experiences and qualifications, as well as their opinion on the participation of pharmacists in veterinary medicine. The interview utilized an open questioning technique and participants could decline to answer any questions. The interviews were recorded and later transcribed for further analysis.

The survey for veterinary students was distributed via Survey Monkey to students at Massey University, New Zealand, undertaking year 4 and 5 of the Bachelor of Veterinary Science (BVSc). The survey consisted of eight questions and took approximately 10 min to complete (Appendix B). The questions focused on the current knowledge the students had about veterinary medicines.

Data consisted of responses to the survey and semi-structured interviews. Quantitative analysis of the data in the survey was performed using Microsoft Excel and Chi-squared (χ^2^) tests were performed to investigate the association between year of study and topic of study. Interviews were transcribed and a thematic analysis performed.

## 3. Results

### 3.1. Veterinarians Views on Relationships with Pharmacisits

The veterinarians interviewed (*n* = 4) had a minimum qualification of a BVSc and were working in urban practices in the Dunedin area of New Zealand. The practice experience ranged from 4 to 30 years and was mainly in the care of companion animals such as cats and dogs. The majority of the veterinarians interviewed do not contact pharmacists on a regular basis. When they did, it was for a specific medicine inquiry. The examples given were a veterinarian contacting a community pharmacist about the availability of insulin glargine for their animal patient because the insulin available to the vet did not have a long duration of action; veterinarians also contacted hospital pharmacists about sourcing chemotherapy medication for their patient. However, they believe that communication with pharmacists is beneficial because pharmacists are able to readily provide them with the information they need.

All of the veterinarians concurred there were areas within their professional where they would like to improve their knowledge. One veterinarian stated that there was a lack of guidance on the use of antibiotics in animals and more specifically, antibiotic resistance and what this means for their animal patients. It was also reported that veterinarians are not taught enough about compounding of pharmaceutical products and the different excipients that make one product different from another, such as the use of preservatives in some medicines. Veterinarians very seldom conducted compounding in their own practices themselves. Their preference was to out-source this task and order compounded medicines via a registered pharmacy in New Zealand that specializes in pharmaceutical compounding.

There were differing views on whether veterinary students are taught enough about medicines during their undergraduate degree. One veterinarian thought that there was not enough information about medicines in the BVSc undergraduate degree, however the students did learn where to locate information when required. To find drug information the following sources were used; Kirk’s Current Veterinary Therapy XV, Index of Veterinary Specialities and Plumb’s Veterinary Drug Handbook. The online resources used by the veterinarian included Veterinary Information Network (http://www.vin.com) and Medsafe New Zealand (http://www.medsafe.govt.nz).

Different factors were considered by the veterinarians interviewed as part of this study before dispensing a medication in-house compared to when writing a prescription to be dispensed elsewhere. A major factor was related to the cost of the medication. Medications that are rarely used or are particularly expensive are less likely to be kept in stock at the veterinarians. In these cases, online veterinary pharmacies were used, where the medicines could be provided to clients at a lower cost. Prescriptions were written on occasion when they were requested by the animal owner, to either use an online pharmacy or to go to another veterinary clinic if the owner was from out of town.

There was a dichotomy of views about the involvement of pharmacists in veterinary medicine. Those that did not support expansion of the role of pharmacists into veterinary medicine provided the reasons that there are differences in the way that medicines are metabolized between humans and animals and therefore some human products are not safe for use in animals. Veterinarians are also able to provide practical advice to animal owners, such as strategies to successfully administer medicines to animal patients, which pharmacists who do not have regular contact with animals would not know. The alternative position was that there were no reservations regarding pharmacist involvement because it occurs in other countries and pharmacists may be able to help with stock issues and easier methods of sourcing medicine that are not often used in the veterinary setting.

With regards to whether additional education would be needed for pharmacists to contribute to veterinary medicine, all but one of the veterinarian believed it would be required. The reasons outlined for the need for further training included the differences in animal physiology and pharmacokinetics in comparison to humans and legalities surrounding medicine choice. 

### 3.2. Views from Veterinary Students

The online survey was distributed to 216 4th and 5th year veterinary students at Massey University in New Zealand with 54 responses (54% from 4th year students and 46% from 5th year students).

**Figure 1:**
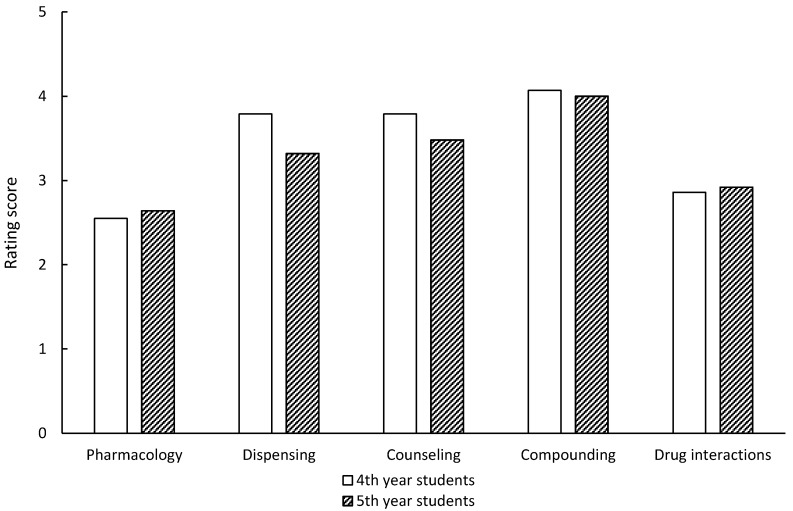
Average rating response by veterinary students about the degree to which they had learnt about aspects of patient treatment (with 1 being a large part of the course and 5 being never).

Veterinary students were asked to state of a scale of 1 to 5 the extent they felt they had learned about five topics relating to patient treatment (Figure 1). There was no apparent difference in response between the 4th and 5th year students for the categories investigated, except dispensing where the 4th year students reported receiving more teaching compared to the 5th year students (χ^2^ (4, *N*= 54) = 17.43, *p* = 0.05). Students report learning most about pharmacology and least about compounding (Table 1).

Students were asked if there were topics relating to patient treatment that they would like to learn more about. Of the 4th year cohort, 21% were interested to learn more about all of the disciplines compared to 12% of the 5th year cohort. Across both years, pharmacology was the topic that >40% of students indicated they would like to learn more about (Table 1). Counseling and compounding were the two areas that students did not indicate a desire to learn more about.

When asked about the likelihood of consulting a pharmacist, the responses showed a trend for general reservation. On a 5-point scale (with 1 being highly likely and 5 being never), the average rating score of both year groups was 3.67 for dispensing, 3.69 for medicines advice and 3.57 for general information. Over 83% of students surveyed were aware that pharmacists are legally able to dispense prescriptions written by veterinarians.

Students were also asked if they had any additional comments to make on the topic. Some students reflected that they would be willing to consult pharmacists about animal medicines.Specific comments were “I would ask a pharmacist if I thought they were open to the idea of helping a vet”, “I would consult a pharmacist in my working life if I knew that was an option” and “I think it would be helpful if pharmacists were available to help give medicines advice”. However, an equal number of students commented that they would be more likely to consults people other than pharmacists if they had enquiries. These people included veterinary colleagues, senior veterinarians, drug representatives, drug companies, veterinary forums or veterinary literature. One reason given for not seeking pharmacist assistance was that they did not know what knowledge pharmacists had about veterinary medicines and would therefore be more likely to consult with veterinary colleagues. However, if they were confident that pharmacists had the required knowledge, they would be willing to consult them.

## 4. Discussion

Animals are an integral part of the discipline of pharmacy in teaching, research and in the practice of delivering healthcare. In teaching the discipline of pharmacy, animals are used in the fundamental sciences of anatomy and physiology that underpin pharmacy. In biomedical research, animals feature as a source of tissue and a number of different animal species are used as disease models. During the new drug approval process, animals feature in the pre-clinical trials that are an essential part of the Food and Drug Administration requirements for new therapeutic agents prior to clinical trials involving human participants. Further, globally there is an interdependence between human and animal health that is described in the One Health framework [14].

New Zealand has a strong reliance on agriculture and a reputation for innovation in the delivery of medicines to animal patients. In New Zealand, veterinary science can only be studied at Massey University in Palmerston North, which is an American Veterinary Medical Association accredited institution. Students graduate with a Bachelor of Veterinary Science after five years of full time study. The Bachelor of Veterinary Science students undertaking their degree at Massey University who were surveyed here had reservations in regard to future consultation with pharmacists should they require additional information about medicines. Concerns were raised by veterinary students about the differences between animals and humans, and whether pharmacists had enough relevant information that would benefit animal patients. The majority of students believe when they are practicing they would be more likely to consult their fellow colleagues or other sources of information for advice about animal medicines rather than pharmacists. The hesitation for collaboration may be attributed to veterinary students being unaware of the knowledge pharmacists have about medicines and the role they play in the healthcare system. Therefore, providing more information about the pharmacy profession and pharmacist’s skills could encourage a further professional relationship.

A limitation to this study is the small number of veterinarians that were included. We have presented this information here to provide a perspective from the veterinary profession and acknowledge that wider consultation is needed. The veterinarians interviewed in this study that were supportive of pharmacist involvement in veterinary medicine quoted functions such as medicine procurement as the main form of involvement. Experience overseas has shown that pharmacists can contribute more than medicine procurement, for example extemporaneous compounding is a task that pharmacists perform for animal patients [15]. The veterinarians interview in the present study commented that the area of their practice where they were least confident is compounding pharmaceutical products. In New Zealand, in a survey of veterinarians who worked with pharmacists to undertake extemporaneous compounding of veterinary medicines, the majority reported that the relationship was beneficial [5]. It was reported in the same study that 33% of veterinarian respondents had worked with pharmacists in the preceding year and all said that they would continue that working relationship [5].

For the veterinarians we spoke to who had reservations about collaboration with pharmacists, in common with veterinary students, their main concern was that pharmacists did not possess the required knowledge about animal medicines. Supplementary education, tailored to the provision of animal medicines would address these concerns. O’Driscoll et al. have similarly noted that in the UK, the involvement of pharmacists with veterinary medicines is not as extensive as it could be and these authors attributed this to pharmacists having insufficient knowledge about veterinary medicines in their undergraduate education [15]. Recently a key article has been published that alerts pharmacists to important considerations when dispensing veterinary prescriptions to avoid medication errors [16]. The authors give examples relevant to contemporary practice where the differences in physiology and pharmacokinetics between animals and humans means that dispensing needs to be tailored to the respective patient. For example, the analgesic paracetamol (acetaminophen) is contraindicated for cats because cats lack the enzymes needed for metabolism and excretion of paracetamol [17]. The resulting toxic metabolites can lead to fatalities if this species receives this medication. 

There are a number of options for pharmacists engaging with education about animal medicines. We acknowledge that there are considerable demands on the time in undergraduate curricula to undertake the essential learning pertaining to human medicine. The addition of further content into undergraduate programmes may be difficult to achieve. Another option that has been presented for pharmacy schools in the United States, an elective course that students can choose to take as part of their studies where the focus is on veterinary therapeutics [18]. An alternative approach post-graduation that can be used to up skill pharmacists in the area of veterinary therapeutics is the provision of continuing education for practicing pharmacists. Online continuing education courses for pharmacists have been established in the United States of America [19]. Currently, the College of Pharmacy at the University of Florida has a continuing education course entitled Veterinary Pharmacy for Practicing Pharmacists that includes; the legal and regulatory aspects relevant to veterinary medicine, descriptions of common disease states for companion animals and the pharmacotherapy options for treatment, public health issues and accurate dispensing of veterinary medicines. There is also the option of specialist veterinary pharmacists within New Zealand whom veterinarians could be directed to for further information about veterinary pharmaceutics. Presently, it is unknown whether or not there would be enough work for specialist veterinary pharmacist in New Zealand. The small population in New Zealand, in comparison to countries such as the Unites States, may not be sustainable for a large number of specialist veterinary pharmacists. Consequently, the viability of a tailored course would need to be carefully considered along with further research to clarify the potential role that a veterinary pharmacist would play in the New Zealand context to benefit animal health.

Ceresia et al. [20] have investigated strategies for the best way to integrate these two professions in the United States. Their research aimed at defining the role of a veterinary pharmacist and ascertaining the most advantageous way of educating such individuals. It was suggested that a residency or fellowship programme would be the most suitable way to gain the required knowledge and experience. An example of a well-established residency programme is that at the North Carolina State College of Veterinary Medicine, where there is a 1 year residency program to prepare participants for certification by the Society of Veterinary Hospital Pharmacists. Similarly, as with the case for pharmacists, there may be opportunities for those in the veterinary profession to learn more about the medicines they use as part of their provision of care. Virtual training opportunities have been suggested by Conrad et al. as a strategy that can be used to experience the complexities of a clinical scenario via a simulations and using active learning principles, the learner can learn to develop the best treatment strategy [14]. In the UK, graduate knowledge of pharmaceutics, drug formulation and compounding are reported to be lacking in veterinary graduates [12]. In the present study the veterinary students who were surveyed identified pharmacology as an aspect of their studies that they found challenging. The students also felt they did not have enough knowledge regarding the compounding, counseling and dispensing of medicines. Specifically, dispensing knowledge was dependent on the year of study they students were completing and more dispensing experience was gained in the final year of the degree. This corresponds to the occurrence of more placement time in the final year where there are more opportunities for the students to see dispensing in a clinical setting. This is in comparison to pharmacy students, who learn about dispensing and compounding from the first year of their pharmacy degree.

Online veterinary pharmacies are taking an increasingly significant place in the animal medicines market. There are a number of online veterinary pharmacies in New Zealand that supply prescription items and supplements for cats, dogs and horses as well as some that have an emphasis on the rural sector with a wider range of animals including cattle, sheep and birds. To purchase prescription medicines from the online pharmacies, clients must first obtain a valid prescription from a veterinarian. New Zealand legislation states that the online pharmacy must have the original signed copy of the prescription before the medicines can be dispensed. Currently, pharmacists in New Zealand are not involved with these online veterinary pharmacies, however there is one registered pharmacy in New Zealand (Optimus Healthcare Ltd.) that specializes in compounding, including veterinary products, and where pharmacists are employed.

## 5. Conclusions

Pharmacists optimize patient health care through collaboration with medical practitioners. There is interest in New Zealand to expand the pharmacist’s role to work with veterinarians to add animal medicines in their scope of practice to enhance treatment for animal patients. It is also acknowledged that appropriate education and training is also needed before pharmacists can contribute to a productive partnership. The wider implications of collaboration between veterinarians and pharmacists is in the application of One Health principles. Through this study, it was identified that an enhanced understanding of the roles that each of the professions performed would assist in the utilization of reciprocal skills.

## Figures and Tables

**Table 1:** Areas identified by veterinary students at Massey University in New Zealand where they would like to learn more about.

Topic	4th Year Students	5th Year Students
Pharmacology	48%	40%
Dispensing	7%	0%
Counseling	0%	8%
Compounding	21%	28%
Drug interactions	31%	24%
All	21%	12%

## Appendix A

Survey questions for veterinarians.

Medicines in veterinary studies and interprofessional relationships.

1. Where did you study veterinary medicine?
2. What is your highest qualification?
3. How long have you been practicing as a veterinarian?
4. Do you specialize in a particular group of animals (e.g., livestock, companion)?
5. Do you have any regular communication with a pharmacist regarding animals under your care?
6. Do you think it could be beneficial to have a better communication between veterinarians and pharmacists regarding care for animal patients?
7. What do you think pharmacists need to know about animal patients in order to support your role as a veterinarian?
8. Can you think of any specific examples where you were required to find out information regarding medicines for your animal patients? What were the sources of information you used? (e.g., textbooks, online, local pharmacists)
9. What factors do you consider when writing a prescription that can be filled elsewhere compared to dispensing the drug at your vet clinic?
10. Do you have any reservations about pharmacists becoming more involved with dispensing vey medicines?
11. Do you think that pharmacists should have more education on medicines for animal patients?
12. Do you think that veterinary students learn enough about medicines for animals in their undergraduate course?
13. Are there any topics about medicines that you would like to improve your knowledge in?
14. Do you have any other comments you would like to make on the topic of our discussions about veterinary medicine and pharmacy?

## Appendix B

Survey questions for veterinary students.

1. What year of your BVSc are you currently undertaking?
2. On a scale of 1 to 5 to what extent have you learned about the following? (1 being a large part of the course, 5 being never)
  - Pharmacology
  - Dispensing
  - Counseling
  - Compounding
  - Drug interactions
3. Are there any areas mentioned above that you would like to learn more about? If yes, please specify.
4. If you have learned about veterinary medicines, what aspects of the course did you find most challenging?
5. Where would you go for extra information on medicines?
  - Internet
  - Textbooks
  - Other—please specify
6. To your knowledge, can pharmacists in New Zealand dispense a prescription written by a veterinarian?
7. On a scale of 1 to 5 how likely would you be to consult a pharmacist for the following? (1 being highly likely, 5 being never)
  - Dispensing
  - Medicines advice
  - Information
8. Are there any other comments you would like to make on this topic?
